# The efficacy of physeal bar resection with guided growth in the treatment of physeal arrest with angular limb deformity

**DOI:** 10.1038/s41598-024-64875-y

**Published:** 2024-06-18

**Authors:** Mohammed Salman Alhassan, Kun Bo Park, Hyun Woo Kim, Hoon Park, Kyeong Hyeon Park

**Affiliations:** 1 Department of Paediatric Orthopaedics, King Faisal general Hospital, Hufuf, Saudi Arabia; 2 Division of Pediatric Orthopaedic Surgery, Department of Orthopaedic Surgery, Severance Children’s Hospital, Yonsei University College of Medicine, 50-1 Yonsei-ro, Seodaemun-gu, Seoul, 03722 South Korea; 3 Department of Orthopaedic Surgery, Gangnam Severance Hospital, Yonsei University College of Medicine, Seoul, South Korea

**Keywords:** Medical research, Paediatric research, Musculoskeletal system, Trauma, Fracture repair, Paediatrics, Therapeutics

## Abstract

Premature physeal arrest can cause progressive deformities and functional disabilities of the lower limbs. This study addressed the outcomes after physeal bar resection with or without guided growth (temporary hemiepiphysiodesis) for the treatment of angular limb deformities. We retrospectively analyzed 27 patients (mean 9 years; range, 3–12 years) who underwent physeal bar resection of the distal femur (15 patients), proximal tibia (3 patients), and distal tibia (9 patients) between 2002 and 2020. Fifteen patients underwent physeal bar resection only (Group A), and the other twelve underwent simultaneous guided growth (Group B). The correction angle (angle change between the preoperative and last follow-up values) was compared and analyzed. The overall mean correction angle was 2.9° (range, − 9 to 18.3°). A total of 12 (45%) patients had a > 5° angular deformity improvement (mean, 9.6°; range, 5–18.3°), 9 (33%) had a < 5° angular change; and 6 (22%) had a > 5° worsening of the angular deformity (mean, 6.7°; range, 5.2–9°). The correction angle in Group B (mean 7.6° ± 6.2) was significantly higher than that in Group A (mean − 0.77° ± 6.3) (P = 0.01). We found six (40%) and zero patients with a > 5° angular deformity increase in Groups A and B, respectively (P < 0.047). The group that underwent physeal bar resection with guided growth showed significantly higher correction angles than the group that underwent physeal bar resection alone. Additionally, none of the patients in the guided growth group experienced an increased angular deformity. Therefore, combining guided growth with physeal bar resection may lead to better outcomes in the treatment of growth arrest with angular deformities.

## Introduction

Physeal injury from fractures, infections, or tumor-like conditions can lead to the formation of a physeal bar and tether bone growth^1–3^. A physeal bar can cause partial physeal growth arrest and progressive angular deformities and functional disabilities^4,5^. Physeal bar resection (PBR) is a promising method for restoring the ability of growth plates. Traditional indications for physeal bar excision include growth plate arrest < 50% of the involved physis and at least 2 years of growth remaining in the involved physis^6^. However, lesions with > 25% of bar formation in the involved physis have a poor prognosis^7^. Many surgical techniques have been introduced for PBR, including fluoroscopy-assisted, arthroscopy, and recently, navigation-assisted physeal bar excision^8–10^.

Limb realignment should be considered in combination with PBR for patients with concomitant angular deformities. Corrective osteotomy is the standard procedure to restore normal limb alignment if the growth plate is closed or close to maturity and can be performed acutely or gradually using external fixation^11^. However, this procedure is invasive, and the indications for corrective osteotomy at the time of bridge resection remain controversial. Recently, guided growth has attracted interest as a less invasive procedure for treating angular deformities in skeletally immature patients. Guided growth has the advantages of being reversible, minimally invasive, and having a lower risk of permanent growth arrest^12^. It provides gradual deformity correction while the implant is fixed with asymmetrical suppression of the target physis^13^, and the suppressed growth can resume when the implant is removed.

There is limited evidence regarding the effects of guided growth combined with PBR. In this study, we aimed to evaluate the outcomes of PBR with or without guided growth procedures and to provide a better understanding of the combination for treating physeal growth arrest with angular deformity.

## Materials and methods

This retrospective study was approved by the Yonsei University Health System, Severance Hospital, Institutional Review Board (IRB No. 4-2022-1529). All study methods conformed to the principles set by the Declaration of Helsinki. The Institutional Review Board of Yonsei University Health System, Severance Hospital waived the requirement for informed consent because of the retrospective observational nature of the study.

### Patients

This retrospective comparative study was conducted in one university teaching hospital. The inclusion criteria were as follows: patients with physeal growth arrest at the lower limb (distal femur, proximal tibia, and distal tibia), patients with angular deformity, and patients who had received PBR using the Langenskiöld procedure^1^. Physeal growth arrest was diagnosed using radiography and computed tomography (CT). Initially, 34 patients were enrolled cohort database from January 2000 to December 2020. Then, we excluded 7 patients with inadequate images or insufficient follow-up (less than 2 years). Finally, 27 patients were enrolled in the present study. Among all patients, those who underwent PBR only were classified as Group A, and those who underwent PBR with guided growth were classified as Group B. All procedures were performed by pediatric orthopedic surgeons.

### Surgical techniques

After general anesthesia was administered, the patient was placed in a supine position on the operating table with a tourniquet applied to the proximal end of the lower extremity. Under fluoroscopic control in multiple projections, a 2.0-mm Kirschner (K)-wire was introduced at the center of the physeal bar. A metaphyseal cavity directed at the bar was created by increasing the diameter of the core reamers and was enlarged with curettes to facilitate visualization. The cavity was then extended into the epiphysis within the bar, and the guiding K-wire was removed. Burrs and/or curettes were used for bar resection until the normal physis was circumferentially visible using fluoroscopy. A 3.5-mm scope was used in three cases to confirm a normal physis. Peripheral physeal bars were removed through a direct medial or lateral approach. The configuration of physeal bars varied widely from round with a smooth perimeter to irregular with projections and dual or satellite bars. The irregular and satellite bars required the removal of more normal physes to ensure complete removal of all arrested areas. After confirming the resection, the defect was irrigated with normal saline and filled with bone cement in 15 patients and bone wax in 12 patients. The interposition material was inserted to fill the cavity left by the physeal bar resection, preventing contact between the adjacent metaphysis and epiphysis. And it was determined according to the surgeon's preference.

Guided growth was performed through a percutaneous epiphysiodesis using a transphyseal screw (PETS) and tension-band plate (TBP). The PETS was used in 2 cases of lateral ankle epiphysiodesis in which TBP fixation was difficult. The TBP was performed in the remaining 10 cases. PETS was performed as described by Metaizeau et al.^14^. A guidewire was inserted obliquely across the metaphysis and into the epiphysis such that the physis crossed a zone comprising one-third to one-fourth of the physeal width. On the lateral view, care was taken to ensure that the guidewire crossed the midpoint of the physis. A partially threaded cannulated screw was inserted percutaneously to confirm the position of the guidewire under fluoroscopy. A TBP with two cannulated screws was placed spanning the growth plate, as previously described^15^. Through a 3–4 cm incision, each plate was placed in a submuscular or subfascial position, with care taken to preserve the periosteum. Fluoroscopy was used to verify the satisfactory plate placement.

Postoperatively, each patient used an immobilizer for 4 weeks to allow wound healing and protect the resection site from pathological fractures. They were then encouraged to begin training the knee and ankle range of motion. At postoperative week 8, the patients were allowed to place the entire weight on the leg. Radiographic data were obtained to evaluate the effect of the operation during follow-up.

### Patient evaluation

The recorded data included patient demographics, physeal bar size, bar configuration^16^, operative procedures, and complications. Preoperative magnetic resonance images (MRIs) or CT were performed. The images were then evaluated using a PACS workstation (Centricity RIS-I 4.2 Plus; GE Healthcare, Milwaukee, USA). Bar size was measured on sagittal and coronal T1-weighted MRIs or CT using electronic calipers. To assess angular deformity, the preoperative and postoperative anatomical lateral distal femoral angle, posterior proximal tibia angle, and lateral distal tibia angle were measured on anteroposterior and lateral radiographic images. Correction angles (angle change between the preoperative and last follow-up values) were compared and analyzed. The preoperative and last follow-up standard entire lower limb radiographs were taken in the anteroposterior views to measure the change of LLD.

### Statistical analysis

Data were analyzed using IBM SPSS Statistics for Windows version 20 (IBM Corp., Armonk, New York, USA). Continuous variables are expressed as mean ± standard deviation, and the Mann–Whitney test was used to analyze the data. Categorical variables are expressed as rates, and the chi-square test or Fisher’s exact test was used to analyze the data. Results with a P value < 0.05 indicate statistically significant difference.

### Ethical approval

This retrospective study was approved by the Yonsei University Health System, Severance Hospital, Institutional Review Board (IRB No. 4-2022-1529). The Institutional Review Board waived the requirement for informed consent because of the retrospective observational nature of the study.

## Results

In total, 27 patients were included in the study. The mean age at the time of surgery was 9 years (range, 3–12 years). This study included 16 male and 11 female patients. The location of the physeal bar was the distal femur in 15 (56%), proximal tibia in 3 (11%), and distal tibia in 9 (33%) patients. The average size of the physeal bar was 13% of the entire physis (range, 5–47%). The mean follow-up period was 59 months (range, 24–158 months). There were 15 and 12 patients in Groups A and B, respectively. Demographic factors, such as age, sex, involved physeal bar, type of bar, and follow-up period, were similar between the two groups, except for the bar location. The patient demographic data are shown in Table 1.

**Table 1:** Demographics data in the study participants.

	Group A (n = 15)	Group B (n = 12)	P-value
Age, mean ± SD (years)	8.9 ± 3	8.3 ± 2	0.49
Sex			1
Male	9 (60%)	7 (58%)	
Female	6 (40%)	5 (42%)	
Physeal bar location			0.02
Distal femur	6 (40%)	9 (75%)	
Proximal tibia	3 (20%)	0 (0%)	
Distal tibia	6 (40%)	3 (25%)	
Percent of involved physeal bar ± SD (%)	14.5 ± 11	10.7 ± 5	0.49
Type of physeal bar			1
Central	6 (40%)	4 (33%)	
Peripheral	9 (60%)	8 (67%)	
Period of follow-up	60.7 ± 41	60 ± 42	0.87

SD, standard deviation.

P < 0.05 was considered statistically significant.

### Angular deformity correction following physeal bar resection

The overall mean change in angular alignment following physeal bar resection was 2.9°, ranging from 18.3° of improvement to 9° of worsening during the follow-up period. Among the patients, 12 (45%) had a > 5° gradual angular improvement (mean, 9.6°; range, 5–18.3°), 9 (33%) had a < 5° change and were considered unchanged, and 6 (22%) had a > 5° worsening of the angular deformity (mean, 6.7°; range, 5.2–9°).

The angular deformity correction following physeal bar resection was significantly higher in Group B (mean 7.6 ± 6.2°) than in group A (mean − 0.77 ± 6.3°) (P = 0.01). There were eight (66.7%) patients (mean, 10.7°; range, 5–18.3°) with a > 5° angular deformity improvement in Group B and four (26.6%) patients (mean, 7.1°; range, 5–11.9°) in Group A, and the difference between the groups is statistically significant (P = 0.047). Moreover, six (40%) (mean, 7°; range, 5.2–9°) and zero patients had a > 5° worsening of the angular deformity increase in Groups A and B, respectively, with statistically significant difference between the groups (P = 0.047). We also found four (33.3%) patients with a > 10° angular deformity improvement in Group B and one patient in Group A; the difference was statistically significant (P < 0.01). Angular correction of the distal femur was significantly higher in Group B than in Group A (Table 2).

**Table 2:** Mean change of angular alignment by physeal bar location.

Physeal bar location	Group A (n = 15)	Group B (n = 12)	P-value
Distal femur	− 0.91° ± 6.2 n = 6	7.2° ± 6 n = 9	0.04
Proximal tibia	2.9° ± 10.6 n = 3		
Distal tibia	− 2.1° ± 3.7 n = 6	8.6° ± 7.9 n = 3	
All patients	− 0.77° ± 6.3	7.6° ± 6.2	0.01

P < 0.05 was considered statistically significant.

### Leg length discrepancy following physeal bar resection

**Figure 1:**
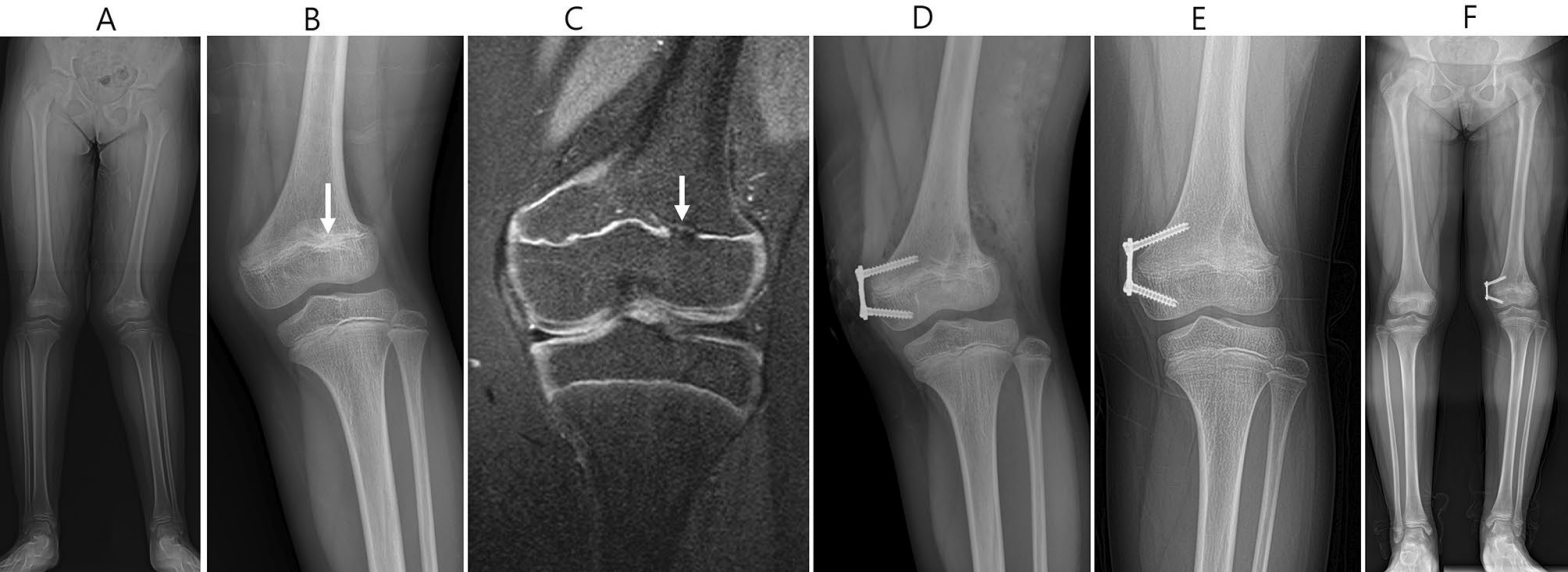
A typical case of physeal bar resection combined with guided-growth (Group B). Preoperative radiographs of the whole leg (WL) and anterior–posterior (AP) in the weight bearing position (**A** and **B**), coronary view of magnetic resonance images (**C**) with the physeal bar (white arrow), and postoperative AP radiograph (**D**) of a 9-years-old boy; the AP and WL radiographs of the patient after 2 years.

**Figure 2:**
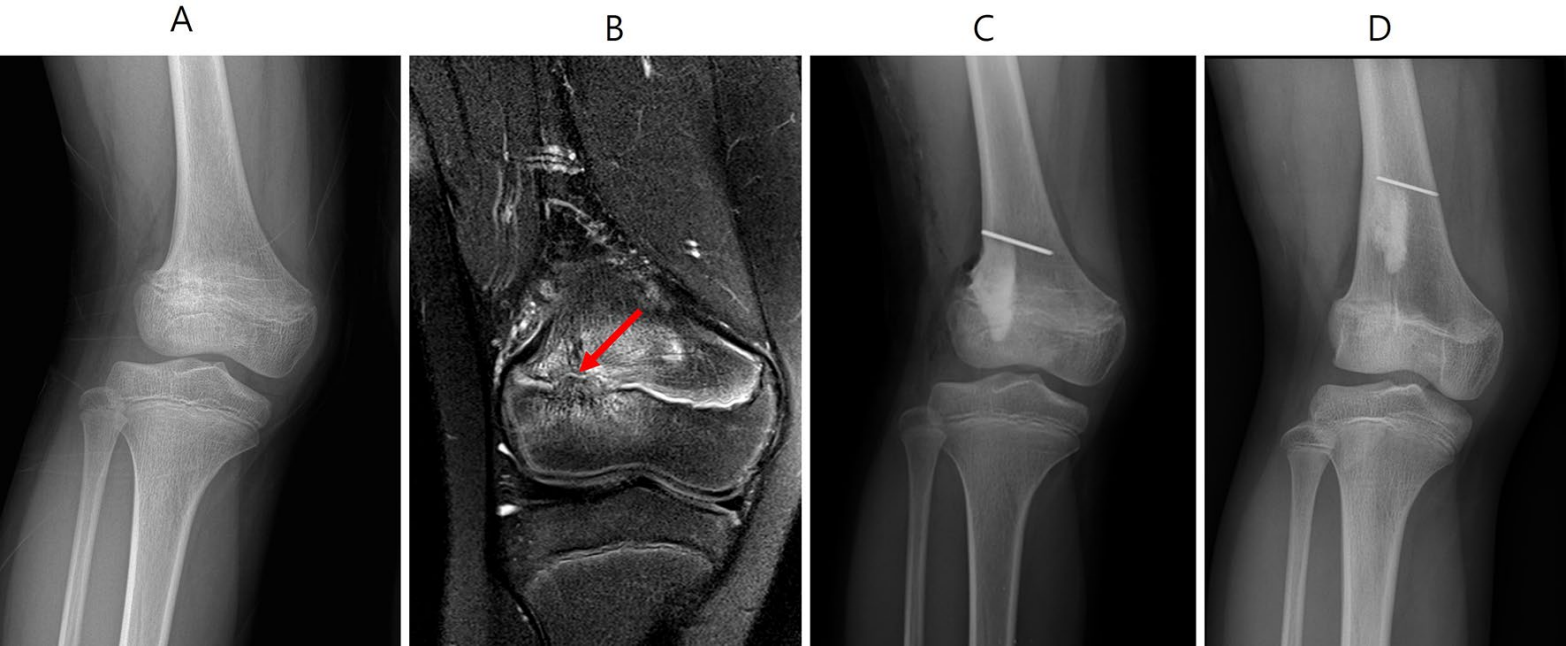
A case of only physeal bar resection (Group A). Preoperative radiographs of knee anterior–posterior (AP) in the weight bearing position and magnetic resonance images (**A** and **B**), with the physeal bar (red arrow), and postoperative AP radiograph (**C**) of an 8-years-old girl. 2 years later, the limb length grew, but the angular deformity did not improve.

Including all patients, the mean LLD at last follow-up was − 1.5 cm (range, 0 to − 5.2 cm) with the affected lower extremity usually shorter than the control limb. The mean change of LLD after physeal bar resection was increased by 3.4mm, ranging from 11mm of improvement to 22 mm of worsening during the follow-up period. There were 12 (44%) patients with LLD > 10 mm and 6 (22%) patients > 20 mm before surgery. After surgery, we found that 11 (41%) patients had a change of < 5 mm and were considered unchanged. Seven (26%) patients had improvement of LLD > 5 mm, averaging 7.3 mm (range, 5–11 mm), and 9 (33%) patients became worse with LLD > 5 mm, averaging 14.1 mm (range, 6–22 mm). The mean change of LLD was not significantly different between the two groups (P = 0.83) (Table 3). No postoperative fractures, infections, or intraoperative complications such as neurovascular injuries were observed. A typical example is shown in Figs. 1 and 2.

**Table 3:** Mean change of LLD by physeal bar location.

Physeal bar location	Group A (n = 15)	Group B (n = 12)	P-value
Distal femur	7.4 mm ± 11.13 n = 6	11.8 mm ± 12.01 n = 9	0.394
Proximal tibia	− 3 mm ± 2.64 n = 3		
Distal tibia	0.71 ± 4.34 n = 6	− 3.12 ± 5.76 n = 3	0.240
All patients	2.65 mm ± 8.38	4.33 mm ± 11.92	0.829

LLD, leg length discrepancy.

P < 0.05 was considered statistically significant.

## Discussion

This study provided valuable insights on the treatment of angular limb deformities resulting from growth arrest, specifically, premature partial physeal arrest. Growth arrest can lead to progressive deformities and functional disabilities of the lower limbs, highlighting the importance of effective treatment options^4,5^. This retrospective analysis of 27 patients who underwent PBR of the distal femur, proximal tibia, or distal tibia shed light on the outcomes and benefits of combining PBR with guided growth.

The findings of this study suggested that simultaneous PBR and guided growth result in a significantly higher correction angle than PBR alone. This indicated that the combination treatment approach was more effective in addressing angular deformities caused by growth arrest. The correction angle represents the degree of improvement in angular deformity, with a higher correction angle indicating a more successful outcome. In this study, patients who underwent PBR with guided growth had a mean correction angle of 7.6°, whereas those who only underwent PBR had a mean correction angle of − 0.77°.

Furthermore, our study demonstrated that the combination treatment approach resulted in a complete absence of angular deformity increase exceeding 5°, whereas six cases of increased angular deformity were observe in the other group. This suggests that guided growth plays a crucial role in preventing the worsening of angular deformities and helps to achieve better overall outcomes.

Literature on the outcomes of combining PBR and guided growth is limited. One successful case was reported by Loraas and Schmale^17^, involving an 8-year-old boy with distal femoral physeal arrest after infection, and endoscopic-assisted PBR was combined with guided growth. Similar principles have been applied to the distal tibial physis. Fu et al.^18^ reported the outcomes of 45 patients with posttraumatic ankle varus deformity who underwent PBR and hemiepiphysiodesis. The median preoperative ankle varus angle was 20°, which was decreased to 5°at the final follow-up. Among the patients, 69% had a 10° decrease in ankle varus angle, indicating an effective procedure. Recently, Masquijo et al.^19^ reported the outcomes of a surgical procedure involving distal femoral PBR and guided growth to treat angular limb deformities caused by growth arrest in the distal femur. Four of five patients achieved total correction within an average of 14.3 months. In our series, 8 (66.7%) of 12 patients who underwent PBR combined with hemiepiphysiodesis had improved angular deformity of the affected limb. These findings suggest that a combination of PBR and guided growth is a promising option for correcting angular deformities associated with physeal arrest.

The results of the present study had important clinical implications. Physicians treating patients with angular limb deformities resulting from growth arrest should consider the combination of physeal bar resection and growth modulation as a viable treatment option. This approach offers advantages in improving correction angles and reducing the risk of worsening angular deformity. These findings emphasize the importance of guided growth in the surgical management of growth arrest-related deformities.

However, this study had several limitations. First, this was a retrospective analysis with a relatively small sample size, which might have affected the generalizability of the findings. Second, although the demographics between the two groups are similar, the physeal bar size and location are not the same. Third, the interposition materials inserted after physeal bar resection and the guided growth method were inconsistent, which may have influenced the results. Although this study had limitations, it provides valuable insights into the surgical management of growth arrest-related deformities and underscores the importance of considering guided growth as a treatment option. Further research with larger sample sizes and comparative studies are warranted to validate these findings and determine the superiority of the combination treatment approach.

In conclusion, this study highlighted the potential benefits of combining PBR with guided growth for treating angular deformities caused by physeal growth arrest. These findings demonstrated that the combination treatment approach leads to significantly higher correction angles and a reduced risk of worsening angular deformities compared to PBR alone. These results suggest that the addition of guided growth to physeal bar resection can improve outcomes and prevent further deformity progression.

## Data Availability

The datasets generated during and/or analysed during the current study are available from the corresponding author on reasonable request.
